# Sclerosing diseases of the skin

**DOI:** 10.1111/ddg.15835

**Published:** 2025-10-01

**Authors:** Yasamin Kalantari, Katharina Meier, Kamran Ghoreschi, Maria Kinberger, Farzan Solimani

**Affiliations:** ^1^ Department of Dermatology Venereology and Allergology Charité – Universitätsmedizin Berlin Berlin Germany; ^2^ Berlin Institute of Health at Charité – Universitätsmedizin Berlin BIH Biomedical Innovation Academy BIH Charité Clinician Scientist Program Berlin Germany

**Keywords:** morphea, skin fibrosis, Systemic sclerosis

## Abstract

Sclerosing skin diseases comprise a group of distinct dermatological conditions characterized by fibrotic changes that may severely impair patients’ quality of life. These conditions often present with cutaneous manifestations and, in some cases, may extend to extracutaneous tissues, potentially resulting in significant morbidity and mortality. This review explores the current understanding of the four most prevalent sclerosing skin diseases – localized scleroderma (morphea), systemic sclerosis (SSc), scleromyxedema, and scleredema adultorum of Buschke – as well as sclerotic conditions induced by external agents. Here, we discuss the pathophysiology, clinical characteristics, and disease course of these entities. In addition, diagnostic tools and treatment options are addressed in detail.

## INTRODUCTION

Sclerosing diseases of the skin encompass distinct dermatologic conditions characterized by fibrotic alterations. These conditions range from localized forms, such as morphea, which primarily affect the skin and subcutaneous tissue, to systemic sclerosis (SSc), a complex autoimmune disease that may involve multiple organ systems – including the lungs, heart, and gastrointestinal tract – and can result in significant morbidity and mortality.^1^, ^2^, ^3^, ^4^, ^5^

These diseases may significantly affect patients’ quality of life and pose diagnostic and therapeutic challenges due to their heterogeneity. Recent advances in disease management and a growing focus on targeted therapies have improved clinical outcomes for affected patients.^5^ In this context, one of the most recent consensus statements on the diagnosis and treatment of sclerosing skin diseases, published by Knobler et al.,^1^ provides clinicians with a detailed overview of first‐ and advanced‐line therapies.

In this review, we discuss current knowledge on the four most common sclerosing skin diseases: localized scleroderma (morphea), systemic sclerosis (SSc), scleromyxedema, and scleredema adultorum of Buschke

## LOCALIZED SCLERODERMA (MORPHEA)

### Clinical presentation and subtypes

Localized scleroderma, also known as morphea, is a chronic autoimmune disease of the connective tissue characterized by abnormal collagen deposits that lead to thickening and hardening of the skin.

Although morphea primarily affects the skin, it may also extend to deeper structures such as the subcutaneous tissue, fascia, muscle, and even bone.

Although morphea primarily affects the skin, it may also extend to deeper structures such as the subcutaneous tissue, fascia, muscle, and even bone.^1^, ^2^, ^3^, ^4^, ^5^ In such cases, complications such as joint contractures, limited range of motion, or growth abnormalities in children, which may include a discrepancy in limb length, may occur. A transition from morphea to systemic sclerosis, which is often feared by patients, is not possible.

Morphea can be categorized into different subtypes based on the clinical presentation and the structures affected. According to the published guideline by Kreuter et al.⁵, the following classification is applied (Table 1):

**TABLE 1:** Clinical subtypes of morphea.

Limited type	Plaque morphea
	Guttate morphea
	Atrophoderma of Pasini and Pierini (Superficial morphea)
	Deep morphea
Generalized type	Generalized Morphea
	Disabling pansclerotic morphea
Linear morphea	Linear morphea of the extremities
	*En coup de sabre*
	*Parry‐Romberg* syndrome (Progressive facial hemiatrophy)
	Eosinophilic fasciitis (Shulman syndrome)
Mixed type	

### Limited types

#### Plaque morphea (Figure 1)

Plaque morphea is the most frequent subtype of limited morphea, especially in adults. It presents with oval and erythematous lesions commonly affecting the trunk and groins. Active lesions are characterized by a purple halo, also known as a “lilac ring”. Over time, the central induration increases and takes a whitish or ivory‐colored hue with a shiny surface. The lesions become more atrophic with a tendency to damage hair follicles and adnexal structures leaving hyperpigmentation.^4^, ^6^, ^7^

#### Guttate morphea (Figure 2b,c)

Guttate morphea presents with multiple yellow‐white coin‐shaped lesions on the trunk that are small (less than 1 cm), and shiny. Early lesions might also present as small erythematous macules.^8^

#### Atrophoderma of Pasini and Pierini (Figure 3)

Atrophoderma of Pasini and Pierini (superficial morphea) is a rare subtype that most commonly presents during childhood.^5^ It is characterized by variably sized, symmetrical, round to oval, erythematous or gray‐brown depressions with cliff‐drop borders. The depressions are caused by a loss of connective tissue.^8^, ^9^

#### Deep morphea (Figure 2a)

Deep morphea is the least common variant of limited subtypes of morphea occurring in less than 1% of patients.^5^ It includes fibrosis of deeper layers of the connective tissue such as fat, fascia, and muscle, and usually manifests as painful lesions on extremities.^8^

### Generalized types

#### Generalized morphea (Figure 4)

Generalized morphea is a more severe and extensive form that occurs in approximately 7% to 9% of patients.^6^ It is defined by the involvement of at least three anatomical regions, most commonly the trunk, lumbosacral area and thighs.^5^ The lesions typically present symmetrically and tend to merge into larger plaques.

#### Disabling pansclerotic morphea

This debilitating form of morphea commonly affects children under the age of 14. Rapid progressive deep tissue fibrosis or pansclerosis causes significant morbidity including joint contracture, limb discrepancy and immobility in addition to impaired wound healing.^5^, ^10^

### Linear types

#### Linear morphea of the extremities (Figure 5)

This form typically presents as a single linear and unilateral lesion on the extremities, sometimes following the lines of Blaschko. While mild cases may resolve with residual hyperpigmentation, more severe manifestations can lead to flexion contractures and impaired mobility.^8^

#### En coup de sabre (Figure 6a,b)

In this form, linear morphea leads to atrophic depressions on the head and face, most commonly in the frontoparietal region or along the paramedian forehead, resembling a sword‐like cut (en coup de sabre). This subtype has been associated with complications such as various forms of alopecia and cerebral involvement.^7^, ^8^

#### Parry‐Romberg syndrome (Figure 6c)

Parry‐Romberg syndrome, also known as progressive facial hemiatrophy, is characterized by unilateral atrophy of the underlying soft tissue and bone of the head and face, typically without skin fibrosis. Neurologic, ocular and oral involvement may occur, and the condition often results in marked facial asymmetry. Co‐occurrence with *en coup de sabre* is common and has been reported in up to 40% of cases.^7^, ^8^

#### Eosinophilic fasciitis

Eosinophilic fasciitis, also known as Shulman's syndrome, presents with acute inflammation characterized by painful erythematous swelling and nonpitting edema of the extremities, typically sparing the fingers and toes. This initial presentation progresses to deep fibrosis involving the fasciae and subcutaneous septa, leading to a characteristic *peau d'orange* appearance (Figure 7a) and venous furrowing (groove sign, Figure 7b). Peripheral and tissue eosinophilia are commonly observed in affected patients.

#### Mixed type

The diagnosis of mixed morphea is made if two or more forms of morphea are present. Mixed morphea occurs in 15% of patients and is often a combination of “linear type and plaque morphea” or “linear type and generalized type”.^7^

### Epidemiology

Morphea has a global annual incidence of four to 27 cases per million individuals and is 2.4 to 4.2 times more common in females, particularly among Caucasians.^6^, ^7^ In adults, onset typically occurs in the fifth decade of life, with plaque‐type morphea being the most frequently reported subtype. In the pediatric population, disease onset usually occurs between the ages of 2 and 14, with linear morphea being the predominant form.

### Etiology and pathogenesis

The pathogenesis of morphea remains incompletely understood and is considered to be multifactorial. Specific HLA alleles class I (allele HLA–B*37) and class II (allele DRB1*04:04) are associated with morphea. Morphea‐related alleles show an association with rheumatoid arthritis (RA), autoimmune thyroid disease (AITD), type 1 diabetes mellitus, and multiple sclerosis (MS).^7^, ^11^

In the early inflammatory phase, T‐helper (Th)1 and Th17 cells and their associated cytokines are elevated, whereas in later stages of morphea, a Th2‐dominated response prevails.^12^, ^13^, ^14^ Key cytokines include transforming growth factor beta (TGF‐β), platelet‐derived growth factor (PDGF) and connective tissue growth factor (CTGF). These cytokines and growth factors promote fibroblast activation and proliferation, leading to increased deposition of collagen and extracellular matrix (ECM).^15^

### Diagnosis and assessment

#### Histopathology

For complete histological representation, sampling should include the subcutis. The main histopathologic characteristic is the abundant accumulation of ECM with increased deposition of collagen fibers, fibronectin, and elastin.

For complete histological representation, sampling should include the subcutis.^8^, ^16^ The main histopathologic characteristic is the abundant accumulation of ECM with increased deposition of collagen fibers, fibronectin, and elastin. In the active inflammatory phase, thickened collagen bundles arranged parallel to the skin surface are observed, accompanied by inflammatory infiltrates. The infiltrating cells include lymphocytes, eosinophils, plasma cells and histiocytes, which are located within the collagen bundles, around blood vessels and at the periphery of adnexal structures.^7^ In the later stages, as inflammation subsides, the lesion becomes avascular, with thickened vessel walls and densely packed collagen bundles.

Deep morphea primarily involves the deep connective tissue with significant sclerosis and hyalinization extending into the underlying fascia, adipose tissue, or underlying muscle.^4^, ^17^

In pansclerotic morphea, biopsy samples reveal lymphocytes, plasma cells, and pansclerosis throughout the dermis and panniculus.^18^, ^19^

In eosinophilic fasciitis, the inflammation and fibrosis primarily affect the fasciae and lower subcutis resulting in fascial thickening. In the early phase of eosinophilic fasciitis, the inflammatory infiltrates include monocytes, plasma cells, and eosinophils. In later stages, the amounts of inflammatory cells are reduced with few or no eosinophils.^4^

Although histological changes are characteristic in morphea, biopsies alone do not permit to distinguish between morphea and skin manifestation in systemic sclerosis.

#### Laboratory tests

At initial diagnosis, basic laboratory testing should include a complete blood count with differential, inflammatory markers such as C‐reactive protein and erythrocyte sedimentation rate, as well as lactate dehydrogenase and liver and kidney function parameters. If concomitant myositis is suspected, creatine kinase should be determined. If arthritis or arthralgia symptoms are present, it may be useful to measure rheumatoid factors and CCP antibodies. An antinuclear antibody (ANA) titer should also be assessed. Up to 50% of patients show elevated ANA levels, particularly antihistone antibodies (AHA) or anti‐single‐stranded DNA antibodies, whereas other autoantibodies are detected in fewer than 10% of cases.^13^ Positivity for the aforementioned three autoantibodies has been associated with more severe disease forms, such as generalized and mixed‐type morphea. The presence of two or more of these antibodies increases the likelihood of deep muscle involvement.

However, the decision to perform the aforementioned laboratory tests should be guided by the patient's clinical presentation, and not all parameters may be indicated in every case.

#### Clinical scores

The only validated clinical score for assessing morphea over time is the *Localized Scleroderma Assessment Tool* (LoSCAT). It comprises the *Localized Scleroderma Skin Severity Index* (LoSSI), which measures disease activity on a scale from 0 to 3 at 18 anatomical sites; the *Localized Scleroderma Skin Damage Index* (LoSDI), which assesses disease‐related damage using the same scale and distribution; as well as the *Physician's Global Assessment of disease activity* (PGA‐A) and *damage* (PGA‐D), both based on 100‐mm visual analog scales ranging from 0 to 100.

Other clinical scores have been proposed but are not validated for morphea. These include the *modified Rodnan Skin Score* (mRSS), the *DIET score* (assessing dyspigmentation, induration, erythema and telangiectasia), and the *visual analog scale* (VAS). More recently, the *Morphea Activity Measure* (MAM) and the *Total Morbidity Score* (TMS) have also been introduced.^20^, ^21^

#### Instrumental diagnostics and devices

Magnetic resonance imaging (MRI) can detect clinically occult neurological and musculoskeletal involvement. MRI is strongly recommended in cases of *en coup de sabre* and progressive facial hemiatrophy to evaluate potential central nervous system involvement.^5^ Moreover, in cases of deep morphea or eosinophilic fasciitis where the involvement is deep, MRI is commonly recommended.^5^

To monitor disease activity, in addition to 20‐MHz ultrasonography, other techniques such as reflectance confocal microscopy (RCM), optical coherence tomography (OCT), infrared thermography (IRT), laser Doppler flowmetry (LDF), as well as the use of a durometer and cutometer may be considered.^5^, ^22^, ^23^ These techniques have primarily been utilized to assess the therapy course in clinical trials.

### Differential diagnosis

In morphea patients, a thorough medical history and assessment for other autoimmune diseases is indicated. The anogenital region should be carefully examined since the coexistence of morphea and lichen sclerosus has been suggested in various studies.^5^

There are multiple subtypes of morphea, each with different stages. As a result, there are numerous potential differential diagnoses to be considered. One important differential diagnosis for morphea is Systemic sclerosis (SSc). Morphea and systemic sclerosis (SSc) share common histopathological features; therefore, histopathology is not a reliable method for differentiating between the two diseases.^24^ Patients with morphea do not exhibit Raynaud phenomenon, sclerodactyly, or nailfold capillary abnormalities, which are characteristic features of systemic sclerosis (SSc).^6^ Moreover, morphea typically does not involve internal organ manifestations.

### Treatment

#### Topical therapy

Topical corticosteroids are considered the cornerstone of topical therapy. During the active phase of morphea, daily application of highly potent corticosteroids for up to 1 month or moderately potent corticosteroids for up to 3 months is recommended. Longer treatment courses should be administered intermittently. For solitary lesions, application under occlusion can enhance the efficacy of topical corticosteroids.^5^ Intralesional corticosteroid injections are recommended for the *en coup de sabre* subtype.⁵ Injection of triamcinolone acetonide (10–40 mg), either undiluted or diluted with lidocaine at a ratio of 1:2 to 1:4, into the active lesion border may yield satisfactory results.^4^ Other proposed topical treatments are off‐label and supported by limited data. Topical calcineurin inhibitors,^4^, ^25^, ^26^ imiquimod, and calcipotriol^4^, ^27^ have shown positive effects in small studies and case series.^8^

#### Phototherapy

Ultraviolet (UV) therapy has demonstrated anti‐fibrotic and anti‐inflammatory properties by reducing extracellular matrix (ECM) components through the activation of matrix metalloproteinases (MMPs). UVA‐based treatments (wavelengths 320–400 nm) are particularly effective for deep lesions, whereas UVB‐based modalities (wavelengths 280–320 nm) may be beneficial for superficial lesions.^7^ Another option is Psoralen plus UVA (PUVA). PUVA therapy can be recommended during the early inflammatory phase of the disease. However, due to the increased risk of skin damage and potential long‐term effects associated with higher UV exposure, PUVA is generally not recommended for use in children.^5^, ^28^

#### Systemic therapy

Methotrexate (MTX) is the first‐line systemic treatment option for morphea with severe skin or extracutaneous manifestations. It is recommended that treatment be maintained for at least 12 months to ensure long‐term disease control.^7^ In cases where the disease is highly active and inflammatory, systemic corticosteroids can be added to MTX for a limited period of about 3 months. Monotherapy with low‐dose systemic corticosteroids can only be recommended for eosinophilic fasciitis.^5^

Methotrexate (MTX) is the first‐line systemic treatment option for morphea with severe skin or extracutaneous manifestations.

For patients who are intolerant to or have contraindications for MTX and corticosteroids, mycophenolate mofetil (0.5–2.0 g/day) is considered the second‐line treatment, particularly for recalcitrant or severe cases of morphea.^29^ In more inflammatory forms of morphea, abatacept has emerged as a promising second‐line treatment. It can be used as monotherapy or in combination with MTX, corticosteroids, or mycophenolate mofetil.^5^

#### Future perspective treatments

Recent research is focusing on target‐based treatments with less side effects. Studies have shown promising results with agents targeting cytokines such as tocilizumab and sarilumab (IL‐6‐receptor antagonists), and infliximab (anti‐TNF‐α).^30^ Imatinib (a tyrosine kinase inhibitor), apremilast (a phosphodiesterase [PDE]4 inhibitor), and everolimus (a mTOR inhibitor) have shown efficacy in treating morphea.^30^, ^31^, ^32^

Ongoing trials are investigating the efficacy and safety of IL‐4/IL‐13 inhibitors. Notably, a randomized, controlled phase IIa clinical trial (NCT04200755) is evaluating the efficacy of subcutaneous injections of Dupilumab 300 mg, administered every 14 days, in patients with morphea.

The Janus kinase (JAK)–signal transducer and activator of transcription (STAT) pathway is implicated in the pathogenesis of morphea, and its inhibition has attracted increasing interest as a potential therapeutic approach.^33^ Tofacitinib and Baricitinib, both targeting this pathway, have shown superior efficacy compared to conventional treatments in selected cases.

It has been concluded that STAT4 gain‐of‐function variants lead to disabling pansclerotic morphea and JAK inhibitor ruxolitinib decreases the dermatologic and inflammatory symptoms.^34^

#### Management of residual atrophy

Disfigurement resulting from subcutaneous tissue atrophy in *en coup de sabre* and progressive facial hemiatrophy can have a substantial psychological impact.^35^ To correct the resulting atrophy, various reconstructive approaches may be employed, including autologous fat grafting, dermal fillers – particularly hyaluronic acid (HA) – surgical techniques, porous polyethylene implants, and acellular dermal matrices.

#### Physiotherapy

Physiotherapy is strongly recommended for patients with morphea, particularly those with linear limb, generalized, or pansclerotic subtypes, at a frequency of 1–2 sessions per week. Massage and lymphatic drainage are beneficial in the sclerotic phase. Physiotherapy would enhance the range of motion although is not recommended in the active stage.^7^

Physiotherapy is strongly recommended for patients with morphea, particularly those with linear limb, generalized, or pansclerotic subtypes, at a frequency of 1–2 sessions per week.

### Prognosis

Patients with morphea often exhibit a relapsing–remitting disease course, particularly in the generalized subtype, highlighting the need for long‐term follow‐up.^36^ The prognosis highly depends on the morphea subtype. Almost 50% of patients diagnosed with limited morphea experience disease regression in 2.5 years.^5^ In contrast, generalized, linear, and deep morphea are associated with a longer disease duration of approximately 5.5 years.

With standard‐of‐care therapy administered over 6 to 12 months, most patients experience an improvement in disease activity within the first 3 to 12 months. However, sclerosis typically resolves more slowly, often over a period of 2 to 5 years, while tissue atrophy may persist or even progress despite treatment discontinuation^36^

## SYSTEMIC SCLEROSIS (SSc)

### Definition and epidemiology

Systemic sclerosis (SSc) is a heterogeneous autoimmune disease that affects both the skin and internal organs, with a female‐to‐male ratio of 3–6:1. It most commonly presents in individuals in their 30s to 40s, without a clear racial predilection.^37^

The prevalence of SSc ranges from 7.2 to 33.9 per 100,000 individuals in Europe and from 13.5 to 44.3 per 100,000 in North America. The annual incidence is estimated at 0.6 to 2.3 per 100,000 individuals in Europe and 1.4 to 5.6 per 100,000 in North America.^38^, ^39^

The development of SSc might be influenced by environmental factors in individuals with a genetic predisposition. Endothelial alterations and immunologic disturbances (involving T‐lymphocytes and their associated cytokines) lead to vascular abnormalities, changes in extracellular matrix compounds, and increased collagen depositions.

### Clinical presentation

Systemic sclerosis can present with a wide range of clinical manifestations involving the skin and internal organs. Many patients report fatigue and a general sense of weakness. Raynaud's phenomenon often appears early in the disease course and may precede other symptoms by several years.^40^ Raynaud's phenomenon is often triggered by exposure to cold or emotional stress, though it may also occur spontaneously. It begins with vasoconstriction, causing the skin to appear pale. The resulting reduction in blood flow leads to local hypoxemia, which gives the skin a bluish discoloration. This phase is followed by reactive hyperemia, which may be painful and causes the skin to become red and flushed. Although most commonly affecting the fingers, Raynaud's phenomenon can also involve other areas such as the nose, ears, lips, and nipples.^41^

Another early manifestation of the disease is the development of “puffy fingers” (Figure 8a), characterized by swelling and erythema of the digits.^42^ As the disease progresses, cutaneous sclerosis leads to skin hardening and tightening, resulting in sclerodactyly (Figure 8b).^42^ This process restricts mobility, and at this stage, fully closing the fist is often difficult or even impossible.

Due to recurrent hypoxia, resulting from Raynaud's attacks and sclerosis‐related circulatory disorders and vasculopathy, patients often develop ulcers on the fingers. These ulcers are typically slow to heal and cause significant pain.^43^ Additionally, depressed, pit‐like scars (Figure 8c) frequently develop in the fingertip area, and nail growth may be impaired, with some patients experiencing complete loss of fingernails.

**FIGURE 1:**
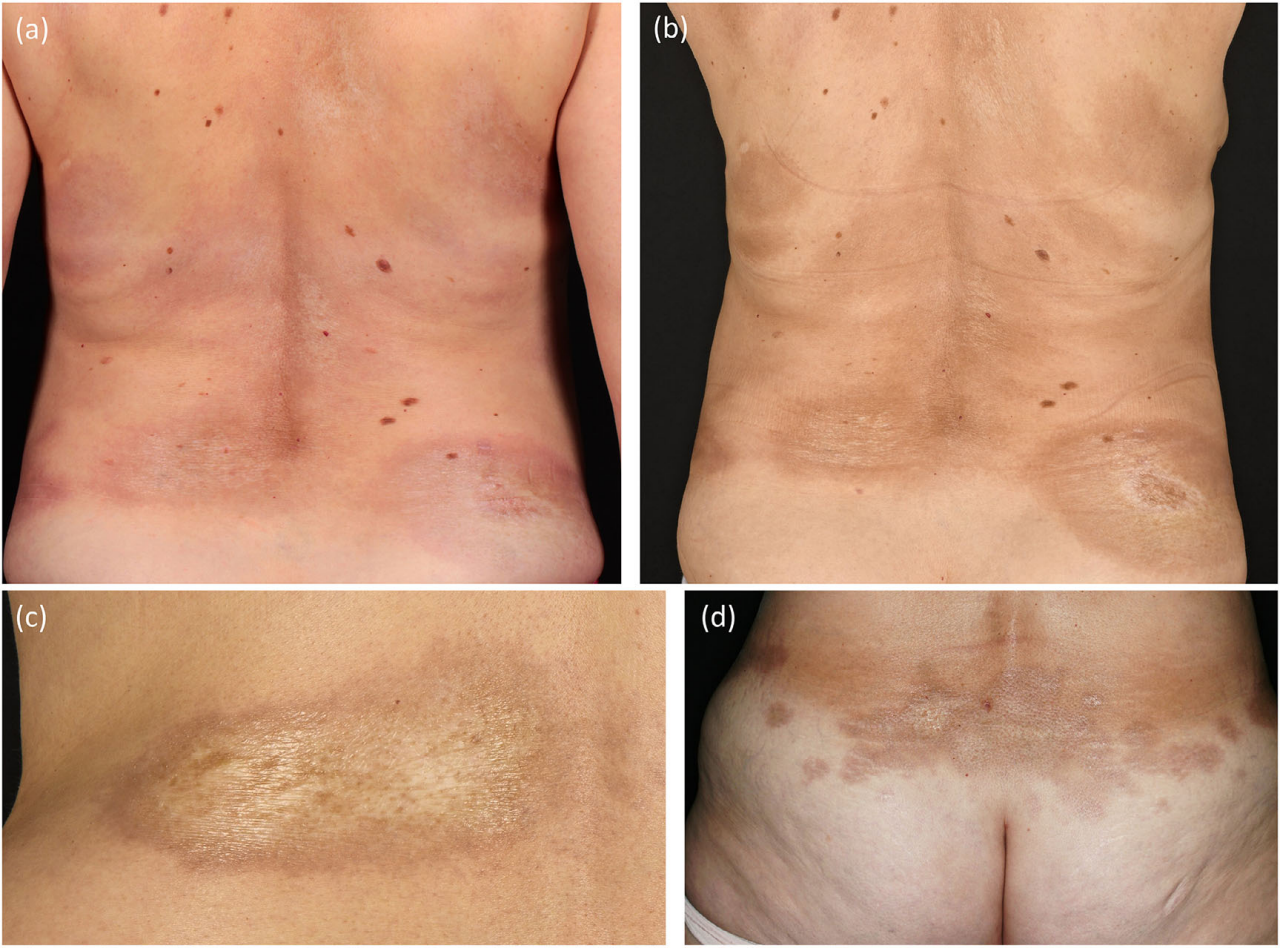
Clinical heterogeneity in plaque‐type morphea. (a) Patient with active plaque‐type morphea with lilac rings; (b) same patient after treatment initiation; (c, d) sclerotic plaques.

**FIGURE 2:**
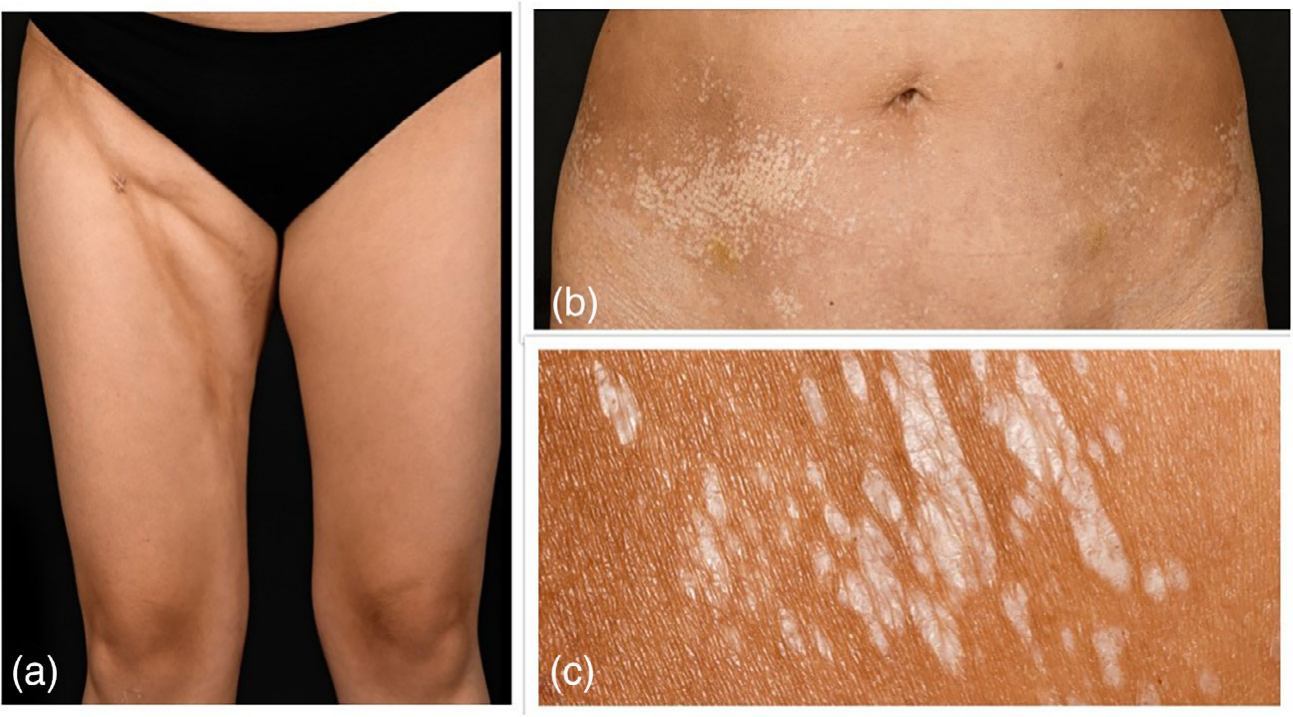
Clinical presentation of two patients. (a) Patient with deep morphea; (b, c) patient with guttate morphea.

**FIGURE 3:**
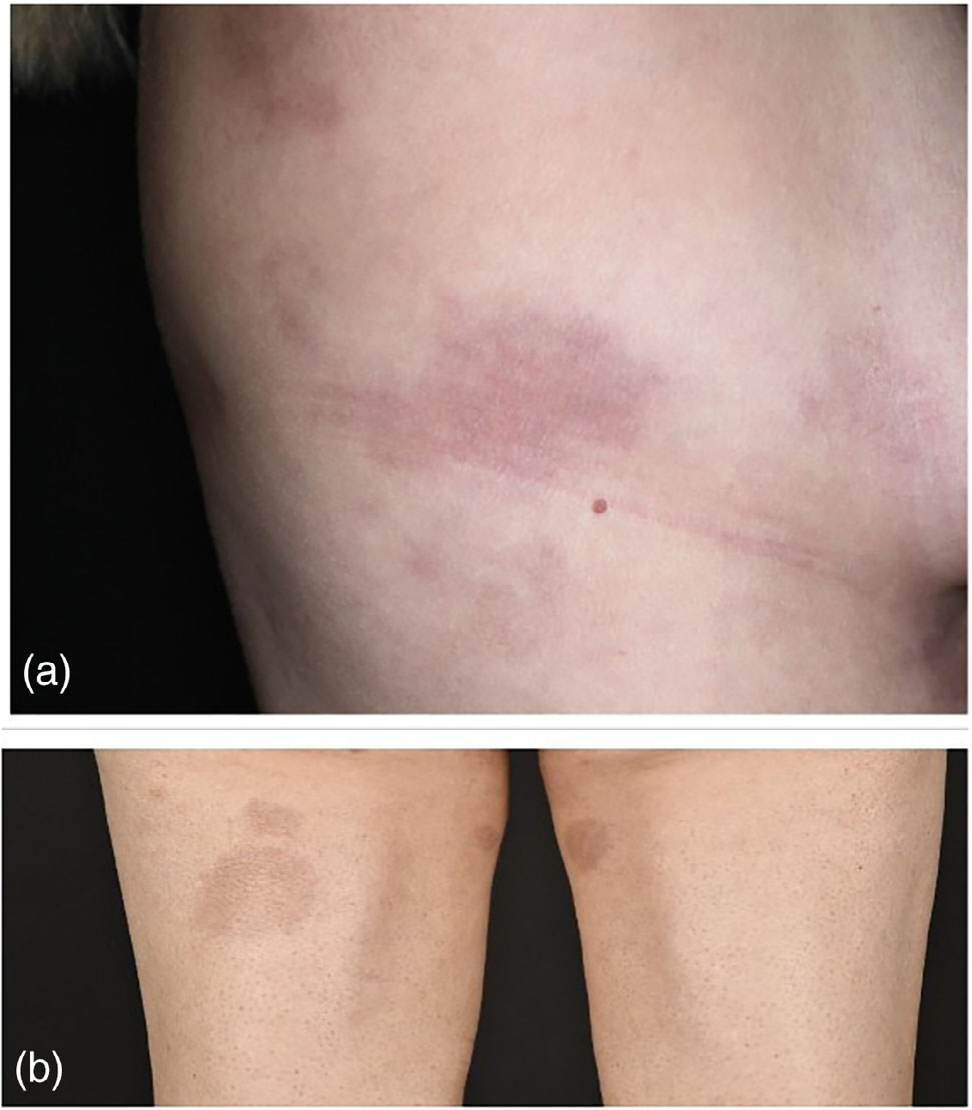
Atrophoderma of Pasini and Pierini in two different patients. (a, b) Clinical presentation.

**FIGURE 4:**
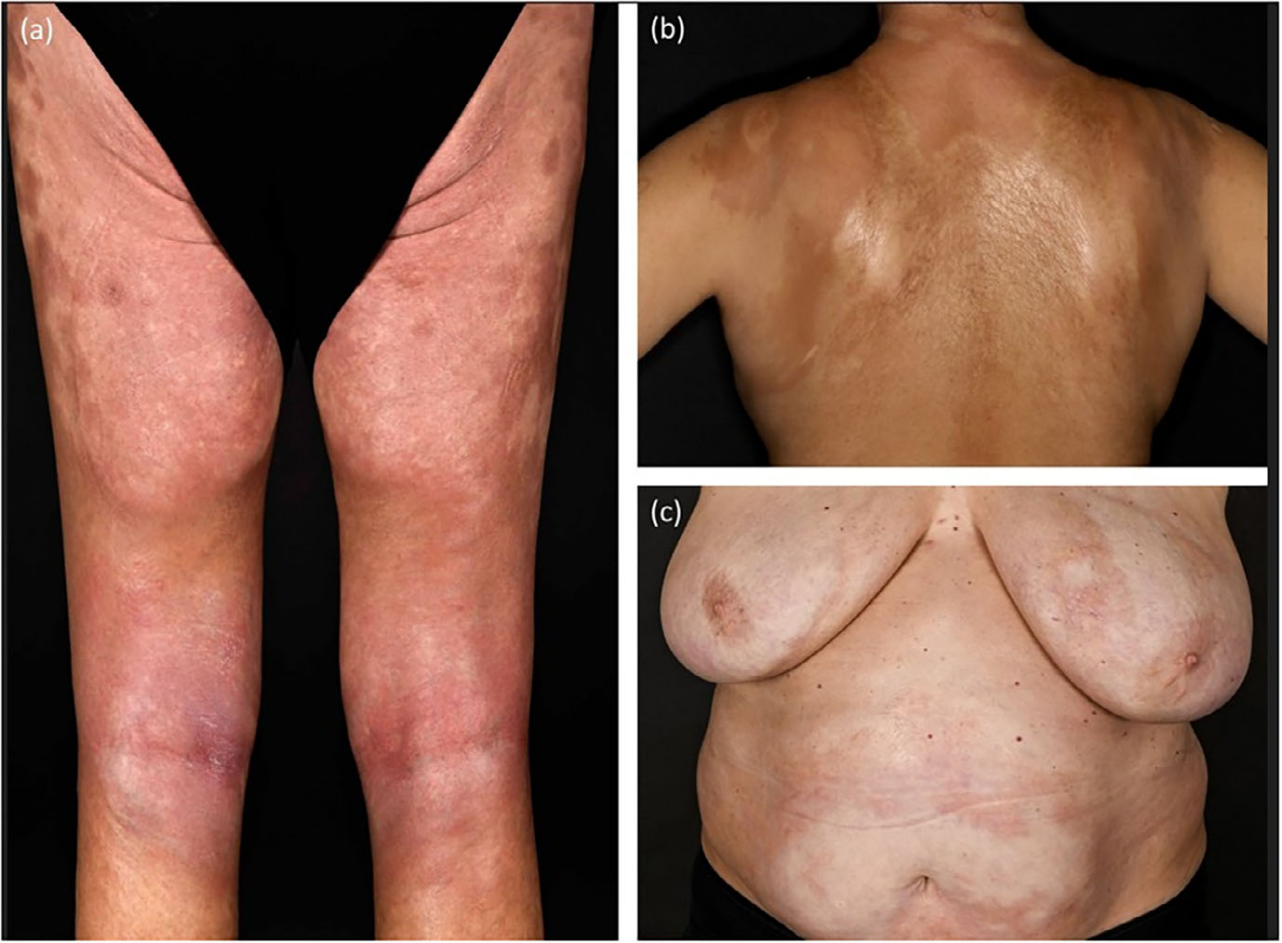
Clinical heterogeneity in generalized morphea. (a) Symmetrical, large sclerotic plaques on the legs; (b) large‐area sclerosis on the back; (c) sclerotic plaques on the upper body with active lilac rings.

**FIGURE 5:**
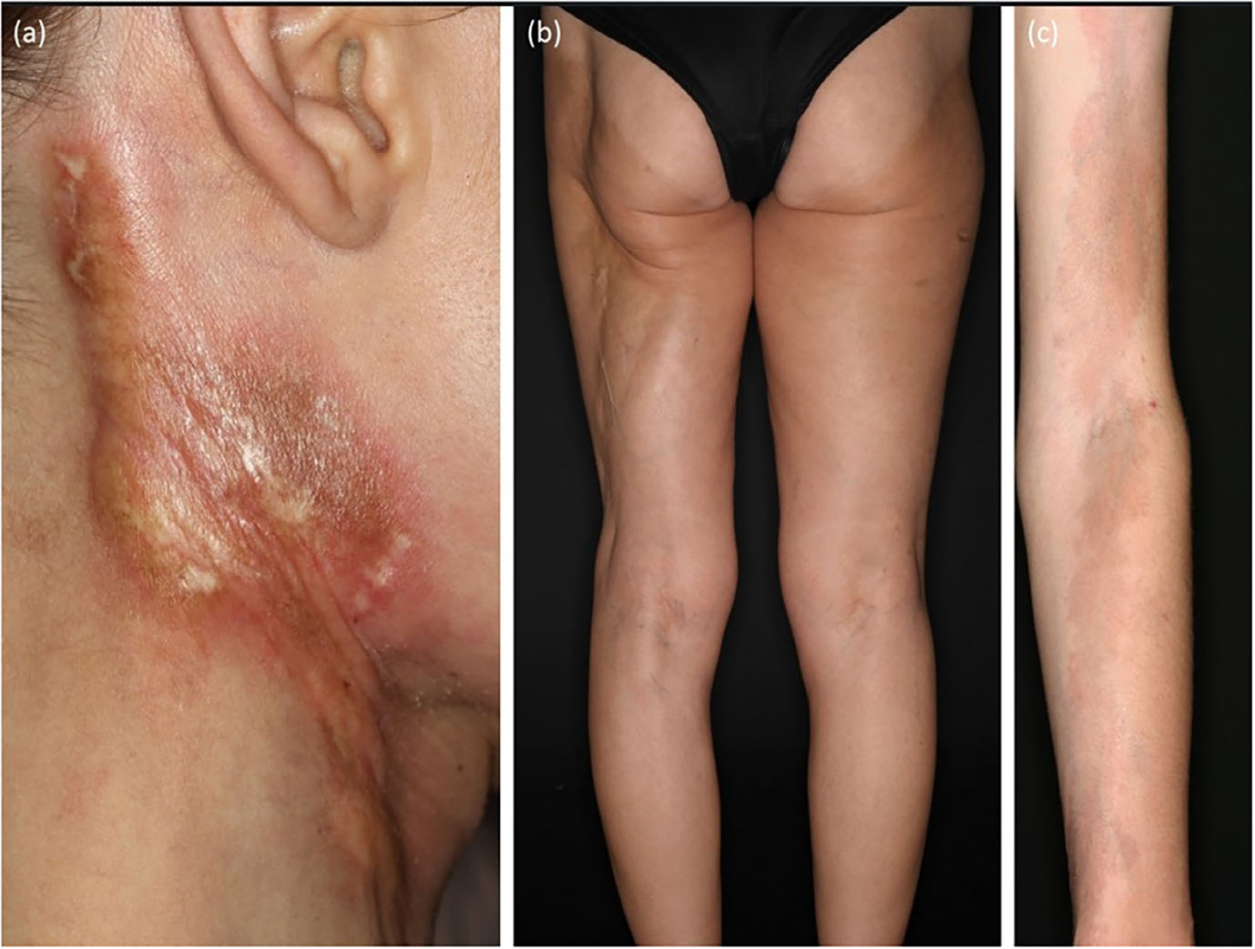
Clinical heterogeneity in linear morphea. (a) Linear morphea on the neck with sclerosed center and marginal lilac ring; (b) deep linear morphea on the left leg; (c) superficial linear morphea on the arm.

**FIGURE 6:**
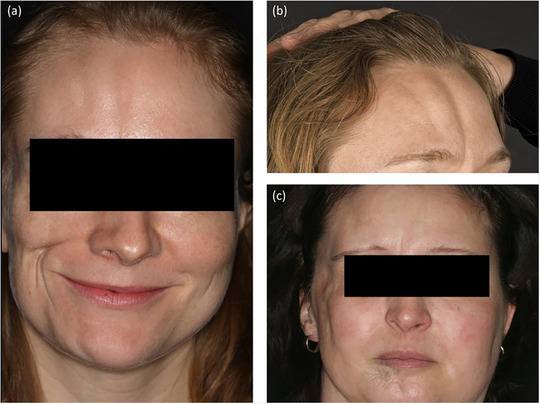
Deep morphea on the face. (a) Parry Romberg syndrome with morphea *en coup de sabre*; (b) morphea *en coup de sabre* with two sabre strokes; (c) Parry Romberg syndrome.

**FIGURE 7:**
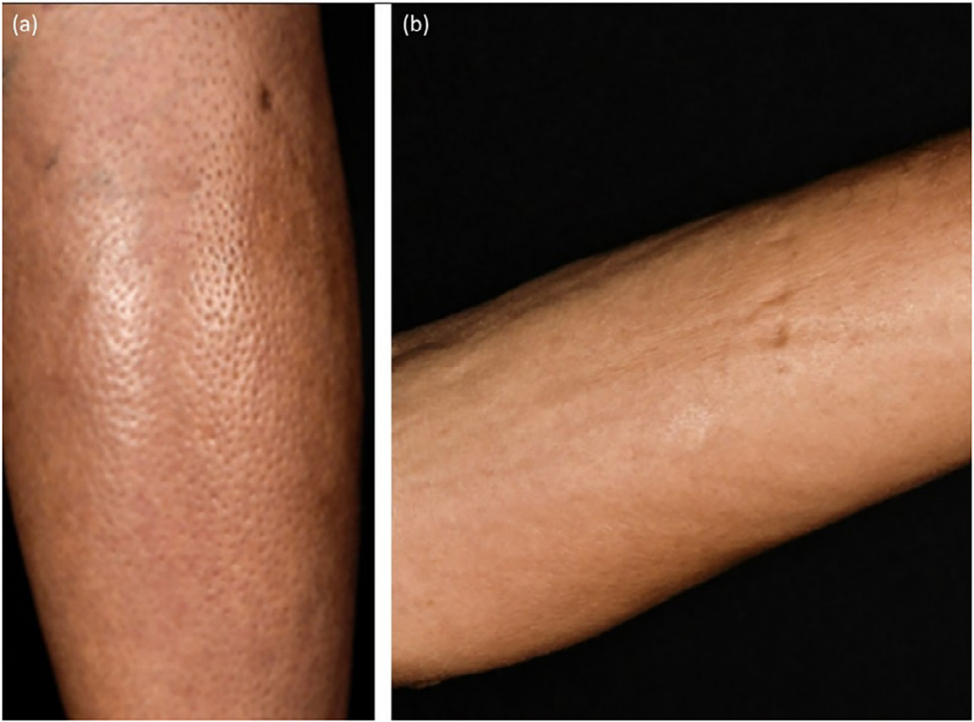
Eosinophilic fasciitis. (a) *Peau d'orange*; (b) groove sign.

Calcinosis, defined as the accumulation of insoluble calcium salts in the skin and subcutaneous tissues, is also commonly observed on the fingers or the extensor surfaces of the extremities.^44^ Calcinosis cutis may also occur in other locations. While some cases remain asymptomatic, others may ulcerate, causing severe pain and markedly limited mobility, especially when lesions develop over joints.

Patients with SSc often exhibit a sclerosis‐related restriction of facial expressions, resulting in what is commonly described as a “mask‐like” appearance. A characteristic feature known as “tobacco pouch mouth” refers to microstomia, accompanied by radial skin folds around the mouth, which limit mouth opening. Frenulum sclerosis may also occur, leading to a shortened frenulum of the tongue.

**FIGURE 8:**
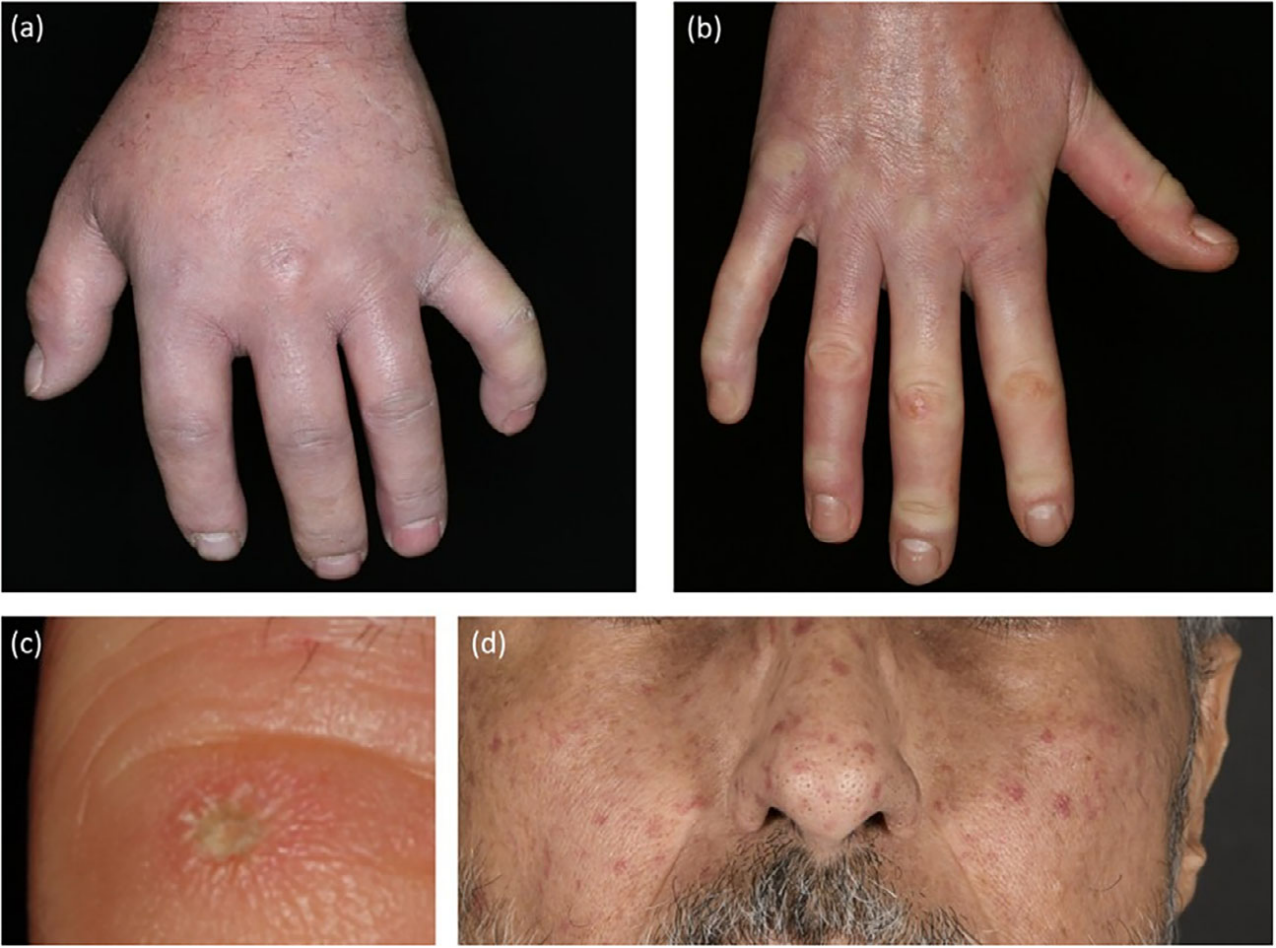
Clinical spectrum in systemic sclerosis. (a) Puffy fingers in a patient with SSc; (b) sclerodactyly with incipient contracture; (c) fingertip pitting scar; (d) facial telangiectasia.

Prominent telangiectasias are another characteristic feature, frequently appearing on the face and often leading to considerable cosmetic concern for patients (Figure 8c).

**FIGURE 9:**
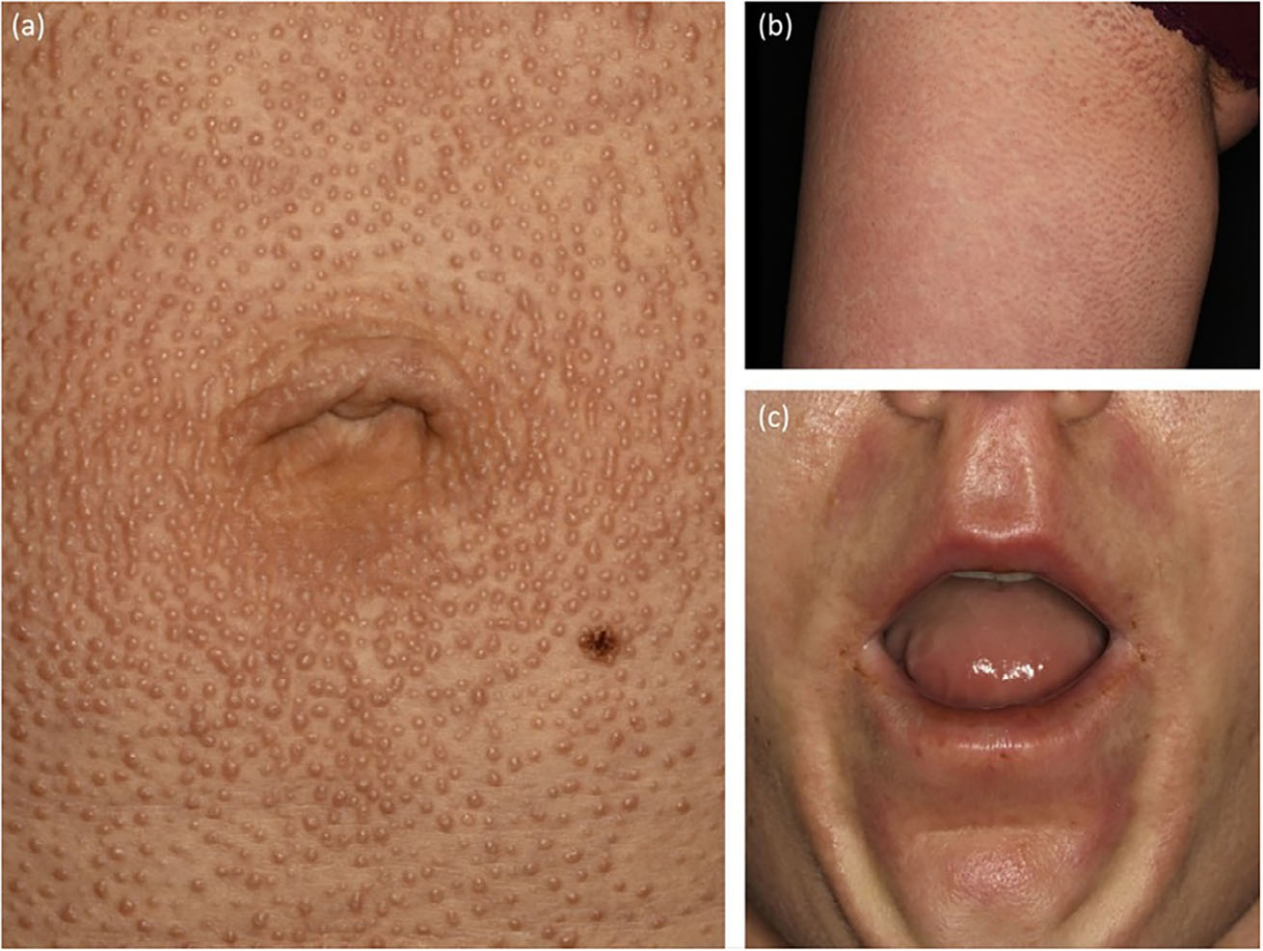
Scleromyxedema. (a, b) Widespread waxy papules; (c) peribuccal skin thickening.

Historically, systemic sclerosis (SSc) has been classified into limited cutaneous SSc (lcSSc) and diffuse cutaneous SSc (dcSSc) based on the extent of skin involvement, according to the classification proposed by LeRoy et al.^45^ The diffuse form is described as a rapidly progressive form of SSc affecting the trunk, extremities and face. Raynaud's phenomenon is an early phenomenon in these patients, usually occurring within a year of the onset of skin changes. Patients are at risk of developing pulmonary fibrosis, and anti‐topoisomerase I antibodies (ATA), also known as anti‐Scl‐70 antibodies, are frequently detected.

In the limited form of systemic sclerosis (lcSSc), skin involvement predominantly affects areas distal to the elbow and knee joints. Patients typically present with long‐standing Raynaud's phenomenon and acral sclerosis. Anticentromere antibodies (ACA) are usually detectable, and there is an increased risk of developing pulmonary arterial hypertension.

With the identification of numerous SSc‐associated autoantibodies that offer insights into disease prognosis and organ involvement, the traditional classification of systemic sclerosis into diffuse and limited forms is now considered outdated, although it remains widely used in clinical practice. Similarly, the term CREST syndrome – formerly used to describe a subtype of limited cutaneous systemic sclerosis (lcSSc) characterized by calcinosis, Raynaud's phenomenon, esophageal dysmotility, sclerodactyly, and telangiectasia – is now considered obsolete.

Musculoskeletal involvement in SSc may manifest as myalgias, arthralgias, or arthritis. Myositis and elevated muscle enzyme levels can also occur, particularly in patients with overlap syndromes.^40^

Interstitial lung disease (ILD) is a common pulmonary complication of SSc, particularly in patients who are positive for anti‐Scl‐70 antibodies.

Interstitial lung disease (ILD) is a common pulmonary complication of SSc, particularly in patients who are positive for anti‐Scl‐70 antibodies.^46^ The pathogenesis involves the recruitment and activation of fibroblasts, leading to an excessive production of ECM. This accumulation progressively replaces normal lung architecture with fibrotic tissue, resulting in lung fibrosis and significant impairment of respiratory function. ILD may follow a rapid and progressive course, potentially leading to respiratory failure. Early clinical signs include a persistent dry cough, exertional dyspnea, and cyanosis.^47^

Pulmonary hypertension (PH) is a heterogeneous condition, and several forms can be associated with SSc, including pulmonary arterial hypertension (PAH), PH due to left heart disease and PH associated with interstitial lung disease.^48^ PAH often occurs in patients with anticentromere antibodies.^40^ This form of PH is characterized by vascular remodeling that leads to fibrotic thickening and progressive narrowing of the blood vessels, which increases pulmonary vascular resistance. The elevated pressure places significant strain on the right heart, often resulting in right heart failure and, in severe cases, death.^49^, ^50^ Patients with anticentromere antibodies often exhibit only mild skin involvement and generally have a less severe disease course. However, this may lead to insufficient screening for pulmonary arterial hypertension (PAH) – a critical concern given the availability of effective treatment options.

Cardiac involvement in SSc may result from myocardial fibrosis or from conduction abnormalities caused by fibrotic changes in the cardiac conduction system.^49^ Myocarditis and pericarditis are also potential manifestations of SSc, although secondary cardiac involvement such as that resulting from pulmonary arterial hypertension (PAH) is far more common. In addition, chronic inflammation in SSc increases the risk of atherosclerosis, which further contributes to cardiovascular complications. Consequently, optimal control of blood pressure and cholesterol levels is essential in the management of these patients.

Renal involvement in systemic sclerosis (SSc) is characterized by endothelial damage, leading to intimal proliferation and narrowing of small afferent vessels. The resulting reduction in renal blood flow triggers activation of the renin–angiotensin–aldosterone system (RAAS). This process can lead to hypertension and renal insufficiency. Mild proteinuria, slightly reduced glomerular filtration rate, or hypertension is therefore often observed in patients with SSc.^51^ However, patients may also develop a severe and life‐threatening renal crisis, which represents the second most frequent cause of death in SSc. This complication occurs predominantly in patients with anti‐RNA polymerase III antibodies, and the risk is further increased by high‐dose corticosteroid therapy.^51^, ^52^ Renal crisis is characterized by acute renal failure, often accompanied by malignant hypertension.

Gastrointestinal involvement is very common in SSc and caused by neural dysfunction, atrophy of the smooth muscles, fibrosis, vasculopathy, impaired regulation of microcirculation and changes in the intestinal flora.^53^, ^54^ A disorder of esophageal motility, particularly in the distal third, which is lined with smooth muscle, often occurs early in the course of the disease. Patients commonly experience this as difficulty swallowing.^54^ Cardia insufficiency contributes to the development of gastroesophageal reflux disease (GERD) in up to 85% of patients with SSc. Moreover, impaired esophageal peristalsis prolongs the retention of gastric acid in the esophagus, further aggravating the severity of GERD symptoms.^55^ Reduced intestinal motility can lead to constipation and bloating; however, diarrhea may also occur as a result of small intestinal bacterial overgrowth (SIBO). Additionally, telangiectasias in the gastrointestinal tract may result in bleeding. In the stomach, particularly in patients with anti‐RNA polymerase III antibodies, gastric antral vascular ectasia (GAVE) can develop, a specific form of vascular ectasia responsible for chronic gastrointestinal bleeding.^56^, ^57^

### Diagnosis and assessment

#### Laboratory tests

Regular basic laboratory tests, including differential blood counts, are essential for monitoring SSc. Elevated acute phase proteins and gamma globulinemia are common findings, though not always present. Routine renal function tests and urinalysis should be conducted to detect proteinuria. Elevated NT‐proBNP levels may suggest cardiac involvement, while severe anemia could indicate gastrointestinal bleeding, for example in cases of GAVE. Monitoring blood lipid levels, glucose, and glycated hemoglobin (HbA1c) is also critical for minimizing the risk of atherosclerosis. Depending on the prescribed medication, additional parameters may be necessary. An autoantibody test should always be performed at the time of initial diagnosis (Table 2).

**TABLE 2:** Detected antibodies in systemic sclerosis and associated clinical phenotypes.^41^, ^42^, ^57^

Antigen	Associated clinical findings
Centromere	Limited cutaneous subtype Digital ulcers PAH
Topoisomerase I (Scl‐70)	Diffuse cutaneous subtype ILD
RNA polymerase III (RNAP3)	Diffuse cutaneous subtype GAVE Renal crisis Malignancy
U3‐ribonucleoprotein (U3‐RNP) / Fibrillarin	Early severe organ involvement Cardiac involvement small bowel dysmotility
U1‐ribonucleoprotein (U1‐RNP)	Overlap syndromes
U11/U12 ribonucleoprotein	ILD
Ku	Limited cutaneous subtype
PM/Scl	Myositis overlap syndromes Arthritis
Th/To	Limited cutaneous subtype ILD PAH Pericarditis
NOR 90	Limited cutaneous subtype
Eukaryotic initiation factor‐2B (eIF2B)	Diffuse cutaneous subtype ILD

*Abbr*.: PAH, pulmonal‐arterial hypertension; ILD, interstitial lung disease; GAVE, gastric antral vascular ectasia

#### Instrumental diagnostics

The *modified Rodnan Skin Score* (mRSS) is a clinical tool used to assess skin involvement in SSc and quantify the extent of skin thickening. The mRSS involves a palpatory examination of 17 anatomical regions, with each region scored on a scale from 0 to 3: 0 indicates normal skin, 1 indicates mild thickening where the examiner can easily make skin folds between two fingers, 2 indicates moderate thickening with difficulty in making skin folds and no wrinkles, and 3 indicates severe thickening with inability to make skin folds between two examining fingers.^58^ The total score is obtained by summing the individual scores, with a maximum possible score of 51 points. The mRSS often correlates with disease activity and the progression of SSc, making it a crucial parameter in both clinical studies and patient management.

The *modified Rodnan Skin Score* (mRSS) is a clinical tool used to assess skin involvement in SSc and quantify the extent of skin thickening.

Capillary microscopy is a valuable tool for both diagnosing and monitoring the activity and progression of SSc.^59^, ^60^ Capillaries, which run parallel to the skin surface at the nail fold, can be evaluated using a video capillary microscope, stereomicroscope, USB microscope, or dermatoscope. Assessment typically focuses on the second to fifth fingers of both hands, with the most reliable results obtained from the fourth and fifth fingers of the non‐dominant hand due to their thinner cuticle. Capillary density, morphology, diameter, and extracapillary changes such as hemorrhages or edema are assessed. Cutolo et al. described three characteristic patterns associated with SSc: early, active, and late.^61^ The early pattern is marked by ectasia at the apexes of the capillary loops. The active pattern features megacapillaries with diameters exceeding 50 µm, microhemorrhages, and moderate capillary loss. The late pattern is characterized by avascular areas resulting from pronounced capillary loss, which may be followed by neoangiogenesis leading to the development of megacapillaries.

To assess lung function, patients with SSc should undergo regular pulmonary function testing, including measurement of diffusion capacity (DLCO). If abnormalities are detected, particularly a decrease in DLCO or forced vital capacity (FVC), a high‐resolution computed tomography (HRCT) scan of the chest should be performed. In high‐risk patients, such as those with anti‐Scl‐70 antibodies, HRCT may be useful at the time of initial diagnosis to establish a baseline assessment.

If pulmonary PAH is suspected, right heart catheterization should be performed to establish a definitive diagnosis. In asymptomatic patients, regular echocardiographic screening is generally sufficient.^62^ An electrocardiogram (ECG) should also be conducted to rule out cardiac arrhythmias.^62^ In cases of suspected myocarditis, cardiac magnetic resonance imaging (cardiac MRI) is recommended.

For gastrointestinal involvement, diagnostic procedures such as esophageal manometry, esophagogastroduodenoscopy (EGD), and colonoscopy may be employed to evaluate disease extent.

#### Diagnostic criteria

In 2013, the *American College of Rheumatology* (ACR) and the *European League Against Rheumatism* (EULAR) established classification criteria for SSc, as summarized in Table 3. A diagnosis can be made when a total score of ≥ 9 points is reached.^63^

**TABLE 3:** The American College of Rheumatology/European League Against Rheumatism criteria for the classification of systemic sclerosis.^63^

Item	Sub‐item(s)	Weight
Skin thickening of the fingers of both hands extending proximal to the metacarpophalangeal joints		9
Skin thickening of the fingers (only count the higher score)	Puffy fingers	2
	Sclerodactyly of the fingers (distal to the metacarpophalangeal joints but proximal to the proximal interphalangeal joints)	4
Fingertip lesions (only count the higher score)	Digital tip ulcers	2
	Fingertip pitting scars	3
Telangiectasia		2
Abnormal nailfold capillaries		2
Pulmonary arterial hypertension and/or interstitial lung disease (maximum score is 2)	Pulmonary arterial hypertension	2
	Interstitial lung disease	2
Raynaud's phenomenon		3
SSc‐related autoantibodies (maximum score is 3)	Anticentromere Anti–topoisomerase I Anti–RNA polymerase III	3

In recent years, the VEDOSS (Very Early Diagnosis of Systemic Sclerosis) criteria were developed to facilitate the earliest possible diagnosis of SSc, even before the disease has significantly progressed.^64^ These criteria are based on the understanding that early detection and treatment can slow disease progression and improve patient outcomes. The VEDOSS criteria include the presence of Raynaud's phenomenon, SSc‐specific autoantibodies, and capillary microscopy abnormalities. These criteria aid in identifying individuals in the very early, unclassifiable stages of scleroderma, before the development of significant skin or internal organ involvement.

#### Differential diagnosis

The diagnosis of SSc is primarily clinical. However, a variety of differential diagnoses must be considered.^65^ These include eosinophilic fasciitis, graft‐versus‐host disease (GVHD), overlap connective tissue diseases, scleromyxedema, scleredema adultorum Buschke, amyloidosis, systemic nephrogenic fibrosis, metabolic disorders such as phenylketonuria, exposure to toxic agents such as silica, and genetic conditions such as Werner's syndrome.

### Treatment

Systemic sclerosis presents with a wide range of symptoms, requiring a multidisciplinary approach. The treatment strategy combines immunosuppressive and vasodilatory therapies, tailored to the severity and clinical presentation of the disease.

SSc presents with a wide range of symptoms, requiring a multidisciplinary approach. The treatment strategy combines immunosuppressive and vasodilatory therapies.

For skin fibrosis, general care is crucial, including avoiding cold and trauma, smoking cessation, moisturizing creams, lymphatic drainage, and physiotherapy. Mild fibrosis can be treated with phototherapy (UVA1 or PUVA), while progressive disease requires systemic treatment with methotrexate or mycophenolate mofetil.^66^

In the management of Raynaud's phenomenon, dihydropyridine‐type calcium channel blockers, such as oral nifedipine, should be considered as first‐line therapy.^67^ Phosphodiesterase (PDE)‐5 inhibitors can also be considered. For acute Raynaud attacks, intravenous iloprost may be considered when oral therapies prove insufficient.^67^

For digital ulcers, intravenous iloprost or PDE‐5 inhibitors are advised. Bosentan should be considered to reduce the formation of new ulcers, particularly in patients who experience multiple ulcers despite treatment with calcium channel blockers, PDE‐5 inhibitors, or iloprost.^67^

Patients with pulmonary involvement in SSc should be treated rapidly with immunosuppressive drugs. Major clinical trials such as SLS‐I^68^ and SLS‐II^69^ have established cyclophosphamide and mycophenolate mofetil as disease‐modifying therapies that show significant improvements in forced vital capacity (FVC) and radiological fibrosis.^70^ Since 2020, nintedanib, a tyrosine kinase inhibitor that blocks VEGF, FGF, and PDGF receptors, has been approved for the treatment of SSc associated interstitial lung disease (ILD).^70^ Autologous hematopoietic stem cell transplantation may be considered for patients with progressive diffuse cutaneous SSc and ILD who do not respond to immunosuppressive therapy.

To treat PAH in patients with SSc, (PDE5 inhibitors and endothelin receptor antagonists are recommended, often as part of combination therapy. Intravenous iloprost is also a therapeutic option. Newer second‐line agents such as selexipag and riociguat may provide additional benefit in the management of PAH.^67^

In addressing gastrointestinal symptoms, proton pump inhibitors (PPIs) should be used to manage gastroesophageal reflux disease (GERD) and prevent esophageal ulcers.^67^ Despite the lack of randomized controlled trials, experts advise the use of prokinetic drugs to manage motility disorders such as dysphagia, early satiety, bloating, and pseudo‐obstruction.^67^ Additionally, intermittent or rotating antibiotics may be utilized to address SIBO by potentially improving the bacterial balance in the gastrointestinal tract.^67^, ^71^

New therapeutic concepts such as anti‐CD20 inhibitors, anti‐IL6 inhibitors or chimeric antigen receptor (CAR) T‐cell treatment are currently being tested.^70^, ^72^

Nevertheless, it remains essential to optimize blood pressure, lipid levels, and blood glucose in patients with SSc.

### Prognosis

Systemic sclerosis is a chronic disease that necessitates long‐term follow‐up. All patients should be assessed for internal organ involvement, particularly of the lungs, heart, gastrointestinal tract, and kidneys. Poorer prognosis is associated with older age (≥ 60 years), the diffuse cutaneous subtype (dcSSc), scleroderma renal crisis, severe dyspnea, reduced forced vital capacity (FVC) and diffusing capacity for carbon monoxide (DLCO) below 70%, anemia, and elevated C‐reactive protein (CRP) levels above 8 mg/l.^73^

## SCLEROMYXEDEMA

### Definition and epidemiology

Scleromyxedema – also referred to as diffuse or generalized lichen myxedematosus, sclerodermoid lichen myxedematosus, Arndt‐Gottron syndrome, or papular mucinosis – is an uncommon primary cutaneous mucinosis characterized by sclerotic skin changes and dermal mucin deposition, most commonly associated with a monoclonal gammopathy.^74^, ^75^ Mucin is a component of the ECM, consisting of acidic glycosaminoglycans produced by fibroblasts. It has a high capacity to absorb water, and in conditions with increased mucin deposition, the dermal connective tissue becomes edematous. Scleromyxedema differs from other variants of lichen myxedematosus, which are confined to the skin, as untreated scleromyxedema may follow a potentially life‐threatening course.

Scleromyxedema –also referred to as diffuse or generalized lichen myxedematosus, sclerodermoid lichen myxedematosus, Arndt‐Gottron syndrome, or papular mucinosis – is an uncommon primary cutaneous mucinosis characterized by sclerotic skin changes and dermal mucin deposition, most commonly associated with a monoclonal gammopathy.

The disease commonly affects middle‐aged individuals, with a mean age of onset around 59 years, and shows no clear predilection for sex or race.^76^

### Etiology and pathogenesis

The most robust data supports the theory that cytokines including IL‐1, TNF‐α, and TGF‐β trigger glycosaminoglycan synthesis and fibroblast proliferation, and are responsible for scleromyxedema pathogenesis. Recent research indicates that chronic Th2‐skewed T‐cell response against an unknown target antigen causes high levels of IL‐4 secretion.^77^

### Clinical presentation (Figure 9)

Scleromyxedema can present with both cutaneous and extracutaneous manifestations (Table 4). The skin is characterized by widespread waxy papules measuring 2–3 mm, typically distributed on the head, neck, trunk, and extremities. As the disease progresses, erythematous and indurated plaques may develop and coalesce, accompanied by skin tightening, sclerodactyly, and restricted mobility of the mouth and joints.^76^, ^78^, ^79^

**TABLE 4:** Cutaneous and extracutaneous features of scleromyxedema.

Organ	Clinical Features
Skin	‐ Widespread firm, waxy, and closely arranged papules (dome‐shape or flat‐top) in lines on head and neck, upper trunk, extremities, and thighs with mucous membranes sparing. ‐ Surrounding skin is shiny and indurated (sclerodermoid) ‐ Presence of erythema, edema, and a brownish discoloration ‐ Involvement of glabella makes the Leonine face ‐ Deep furrowing and deep skin fold (Shar‐Pei) sign ‐ Central depression surrounded by thick skin on interphalangeal joints (doughnut sign) ‐ Pruritus
Nervous system	‐ Peripheral nervous system involvement including carpal tunnel syndrome or peripheral sensory and motor neuropathy ‐ Central nervous system involvement including memory loss, vertigo, gait problems, stroke, seizures, psychosis, or “dermato‐neuro syndrome” ‐ Ocular involvement including corneal opacities and ectropion might be rarely seen
Joints	‐ Arthralgias ‐ Arthritis of the peripheral joints with noninflammatory synovial fluids ‐ Fibromyalgia and severe weakness ‐ In rare cases, dermatomyositis, rhabdomyolysis
Heart	‐ Congestive heart failure ‐ Myocardial ischemia ‐ Heart block ‐ Pericardial effusion
Gastrointestinal system	‐ Dysphagia ‐ Nasal regurgitation
Lungs	‐ Dyspnea ‐ Hoarseness ‐ Aspiration
Kidney	‐ Scleroderma renal crisis‐like acute renal failure

Patients with scleromyxedema commonly present with an IgG monoclonal gammopathy, typically with lambda light chains. Internal organ involvement is possible, including skeletal muscle. Affected individuals may develop muscular, neurologic, rheumatologic, pulmonary, renal, and cardiovascular complications. Clinical manifestations can include esophageal dysphagia, muscle weakness due to myositis, central nervous system encephalopathy, peripheral neuropathy, arthropathies, restrictive or obstructive lung disease, and scleroderma‐like renal involvement, often occurring in conjunction with cutaneous symptoms.

Especially the encephalopathy can have a life‐threatening course. This syndrome begins abruptly with a worsening of skin lesions, a flu‐like prodrome, fever, and seizures, and it can eventuate in an unexplained coma.

### Diagnosis and assessment

#### Laboratory tests

Recommended laboratory evaluations include complete blood count, metabolic panel, muscle enzyme levels, and urinalysis. To detect monoclonal gammopathy – most commonly of the IgG lambda type – serum protein electrophoresis and immunofixation should be performed. Additionally, thyroid function tests are necessary to exclude myxedema secondary to thyroid disease.^80^

#### Instrumental diagnostics

In terms of skin evaluation, high‐resolution cutaneous ultrasonography can be used for skin thickness assessment. Dermoscopy reveals rice grain‐like structures that correspond to papules. Reflectance confocal microscopy shows dermal stellate cells, bright fibers, and dark areas that align with histopathologic features.

Regarding extracutaneous manifestations, tests such as esophageal manometry (for GI involvement) and pulmonary function study (for pulmonary involvement) might be indicated.^80^

#### Histopathology

Histopathologic evaluation is considered the gold standard for diagnosis. The diagnosis of scleromyxedema is based on a triad of *(1)* diffuse mucin deposition (primarily hyaluronic acid) in the upper and mid‐reticular dermis, *(2)* increased collagen deposition, and *(3)* irregularly arranged fibroblasts. An interstitial, granuloma annulare‐like pattern has been reported in approximately 25% of specimens.^80^, ^81^

#### Differential diagnosis

A variety of differential diagnoses must be considered. The most important include systemic sclerosis, scleredema, generalized myxedema associated with severe hypothyroidism, and nephrogenic systemic fibrosis. In addition, other conditions presenting with sclerodermoid skin changes should also be included in the differential diagnosis. The diagnosis of scleromyxedema is supported by the presence of linear papular eruptions, detection of an IgG monoclonal gammopathy, and the characteristic histopathologic triad.^80^

### Treatment

Given the diverse cutaneous and extracutaneous manifestations, a multidisciplinary approach involving rheumatologists, gastroenterologists, and neurologists is essential.^82^ Scleromyxedema has an unpredictable, often progressive course, which necessitates prompt initiation of treatment and regular follow‐up. Long‐term maintenance therapy is typically required, as relapses are common upon discontinuation of treatment. Intravenous immunoglobulin (IVIG) is considered the preferred first‐line therapy for scleromyxedema.^83^, ^84^ Significant improvement in both skin and extracutaneous symptoms, particularly rheumatologic, can often be observed following the initial one or two cycles of IVIG treatment. However, in cases of initial treatment failure or severe disease, additional options such as glucocorticoids (e.g., prednisone, dexamethasone) or other immunomodulatory agents, including thalidomide and lenalidomide, may be considered. The anti‐CD38 monoclonal antibody daratumumab has also shown efficacy in a limited number of studies.^85^, ^86^, ^87^, ^88^

## SCLEREDEMA ADULTORUM BUSCHKE

Scleredema adultorum of Buschke (scleredema) is a rare sclerotic skin disorder, with more than half of affected individuals being under 20 years of age. Although the exact prevalence and incidence are unknown, studies suggest a prevalence of 2.5% to 14.0% among patients with diabetes mellitus.^89^, ^90^ No predilection for gender or race has been observed.

Scleredema can be classified into three types: *(1)* scleredema following infection, *(2)* scleredema associated with hematologic disorders and monoclonal gammopathy, and *(3)* scleredema associated with diabetes mellitus.

Scleredema primarily occurs in association with three clinical contexts and can be categorized accordingly (Table 5): *(1)* scleredema following infection, most commonly after streptococcal pharyngitis and often accompanied by fever; *(2)* scleredema associated with hematologic disorders and monoclonal gammopathy, such as multiple myeloma; and *(3)* scleredema associated with diabetes mellitus (scleredema diabeticorum).^91^

**TABLE 5:** Clinical key points in scleredema adultorum Buschke.

	Underlying conditions	Clinical course	Treatment	Prognosis
Type 1 Scleroedema	Infection, (streptococcal or viral respiratory tract infection)	Sudden onset with fever	Self‐limited (watch and wait) Phototherapy (UVA‐1 phototherapy or psoralen with UVA [PUVA]) If indicated, antibiotics	Good (resolution within 24 months)
Type 2 Scleroedema	Hematological diseases, paraproteinaemia, monoclonal gammopathy, multiple myeloma, amyloidosis	Subtle onset, slowly progressive with chronic course	Treatment of the underlying disease	Poor prognosis with persistent lesions Possible systemic involvement with high morbidity and mortality
Type 3 Scleroedema	Diabetes mellitus	Subtle onset, slowly progressive with a chronic course	Treatment of Diabetes	Poor prognosis Chronic progressive course Systemic involvement

### Pathogenesis

The exact pathophysiology of Scleredema is unknown. It is postulated that various stimuli such as infections (streptococcal), microvascular damage, hypoxia, and drugs along with genetic predispositions cause excessive production of mucin and collagens by fibroblasts.^92^ Scleredema associated with diabetes might result from irreversible glycosylation of collagen, and alterations in collagenase activity.^80^

### Clinical presentation

**FIGURE 10:**
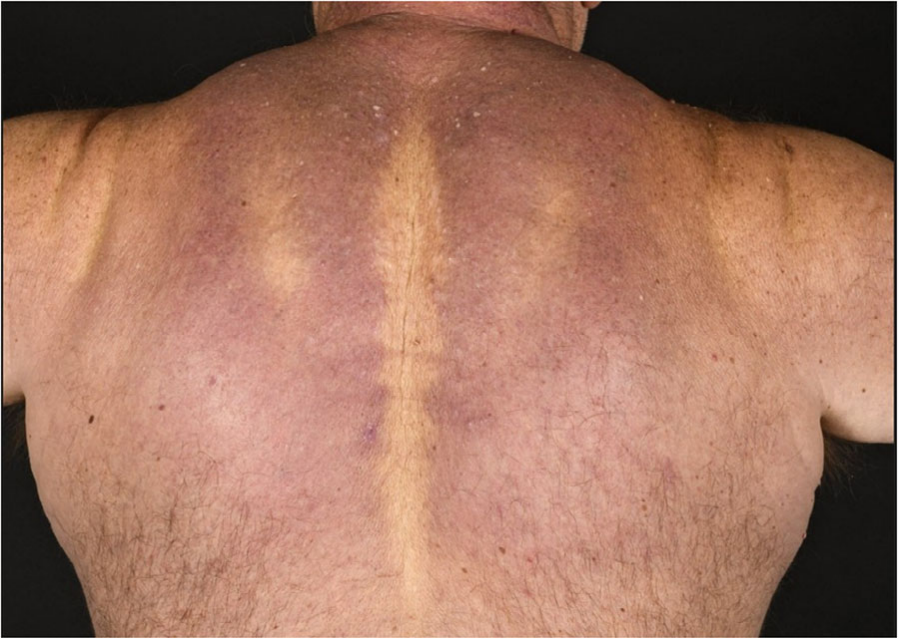
Skin thickening in the area of the neck and upper back in a patient with scleredema adultorum Buschke.

Depending on the type of scleredema, clinical presentations may include both cutaneous and extracutaneous manifestations. Cutaneous features, common to all three types, typically begin with widespread, diffuse, woody skin induration, often accompanied by erythema and a *peau d'orange* appearance. Skin thickening usually starts on the neck or upper trunk and characteristically spares the acral areas (Figure 10). In severe cases, skin stiffness can lead to reduced mobility and functional impairment. Extracutaneous involvement may affect the gastrointestinal, musculoskeletal, ocular, respiratory, and cardiac systems.^93^

### Diagnosis and assessment

#### Laboratory tests

Antistreptolysin O (ASO) titer can be measured to detect recent streptococcal infection. Fasting blood glucose or HbA1c should be assessed to screen for diabetes mellitus. To evaluate for monoclonal gammopathies, recommended tests include complete blood count (CBC), leukocyte differential, serum protein electrophoresis, and immunofixation. Notably, antinuclear antibodies (ANA) are typically negative in scleredema.^80^

#### Instrumental diagnostics

In order to assess skin stiffness, devices such as durometer or ultrasonography could be used.^94^ Based on the severity of the disease and the presence of systemic involvement, additional imaging such as pulmonary function tests (pulmonary involvement), and esophageal manometry (GI involvement) might be indicated.^80^

#### Clinical scores

Given the rarity of Scleredema, no specific clinical score exists for Scleredema. However, mRSS can assess the extent of skin involvement.^95^

#### Histopathology

The epidermis is usually intact and normal. Dermis is three to four times thicker than usual, because of enlarged collagen bundles and mucin‐filled areas (commonly hyaluronic acid) between them. It is worth mentioning that the absence of mucin does not rule out the diagnosis. In Scleredema, fibroblast proliferation is absent, and elastic fibers might be reduced in number.

#### Differential diagnosis

Scleredema may present with clinical features that resemble those of other sclerotic disorders. Key differential diagnoses include systemic sclerosis, scleromyxedema, myxedema, eosinophilic fasciitis, cutaneous amyloidosis, lymphedema, and graft‐versus‐host disease.^80^

### Treatment

In all types, physical therapy is indicated to reduce functional limitations. In cases of severe skin stiffness or systemic involvement, the first line is phototherapy (PUVA, UVA1, and the narrowband UVB) and second line is systemic treatment with MTX (glucocorticoids).^96^ Since the streptococcal‐associated form is usually self‐limiting, systemic therapy is rarely necessary.

### Prognosis

Patients with Scleredema should be followed on a regular basis and should be screened in terms of paraproteinaemia and systemic complications.^97^ In scleredema, symptoms are typically limited to the skin; however, systemic involvement may occur in some cases. Overall prognosis depends on the subtype of scleredema. The best outcomes are seen in individuals with post‐infectious forms, while those with hematologic or diabetes‐associated scleredema generally have a poorer prognosis.

## SCLEROTIC DISEASES INDUCED BY EXTERNAL AGENTS

### Nephrogenic systemic fibrosis

Nephrogenic systemic fibrosis (NSF) is a treatment‐refractory disorder clinically characterized by indurated plaques symmetrically distributed on the extremities and trunk. In severe cases, involvement of internal organs such as the heart, skeletal muscles, and lungs has also been reported.^98^ This condition was first recognized in the early 2000s and has since been associated with exposure to gadolinium‐based contrast agents. Patients with end‐stage kidney disease are particularly at risk of developing NSF following gadolinium administration.^99^ Therefore, the use of certain gadolinium‐based contrast agents – specifically gadopentetate dimeglumine (Magnevist^®^), gadodiamide (Omniscan™), and gadoversetamide (OptiMARK™) – should be strictly avoided in this patient population.

### Toxic oil syndrome

Toxic oil syndrome was caused by the ingestion of aniline‐contaminated, reprocessed rapeseed oil and was observed in Spain during the 1980s. Affected individuals initially presented with maculopapular skin lesions accompanied by flu‐like symptoms. As the condition progressed, the inflammatory process involved the nervous system, lungs, and salivary glands. The skin manifestations evolved from exanthematous lesions to morphea‐like sclerotic changes.^100^

### Eosinophilia‐myalgia syndrome

Eosinophilia–myalgia syndrome was observed in 1989 in the United States and was linked to a contaminated preparation of L‐tryptophan, which many affected individuals had taken as a dietary supplement. The condition presented with systemic symptoms, including myalgias, fever, dyspnea, edema, peripheral eosinophilia, and erythematous skin lesions. Approximately half of the affected patients eventually developed severe induration of the extremities, accompanied by progressive peripheral neuropathy and myopathy.^101^

### Stiff skin syndrome

Stiff skin syndrome (SSS) is a pediatric disorder first described in 1971, typically manifesting at birth or during early childhood.^102^ Clinically, it is characterized by severe induration of the skin and underlying tissues, most prominently affecting the thighs and buttocks. These changes can significantly restrict mobility; however, visceral involvement is absent. Affected individuals often display mild hypertrichosis. SSS is caused by mutations in the *FBN1* gene, which encodes fibrillin‐1. The resulting dysregulation leads to increased TGF‐β activity, which contributes to the characteristic skin induration.^103^

## CONFLICT OF INTEREST STATEMENT

None.
